# Efficacy and safety of incobotulinumtoxinA in post-stroke upper-limb spasticity in Japanese subjects: results from a randomized, double-blind, placebo-controlled study (J-PURE)

**DOI:** 10.1007/s00415-020-09777-5

**Published:** 2020-03-26

**Authors:** Yoshihisa Masakado, Masahiro Abo, Kunitsugu Kondo, Satoru Saeki, Eiichi Saitoh, Andrzej Dekundy, Angelika Hanschmann, Ryuji Kaji, the J-PURE Study Group

**Affiliations:** 1 Department of Rehabilitation Medicine, Tokai University School of Medicine, Kanagawa, Japan; 2 Department of Rehabilitation Medicine, The Jikei University Hospital, Tokyo, Japan; 3 Department of Rehabilitation Medicine, Tokyo Bay Rehabilitation Hospital, Chiba, Japan; 4 Department of Rehabilitation Medicine, Hospital of the University of Occupational and Environmental Health, Fukuoka, Japan; 5 Department of Rehabilitation Medicine I, School of Medicine, Fujita Health University, Aichi, Japan; 6 Merz Pharmaceuticals GmbH, Frankfurt am Main, Germany; 7 Department of Neurology, Tokushima University Hospital, Tokushima City, Tokushima, Japan

**Keywords:** IncobotulinumtoxinA, Botulinum neurotoxin type A, Japan, Spasticity, Upper limb, Stroke

## Abstract

**Background:**

Upper-limb spasticity frequently occurs after stroke and there is a clinical need for more effective therapies. The Phase III J-PURE study assessed the efficacy and safety of incobotulinumtoxinA up to 400 U for post-stroke upper-limb spasticity in Japan.

**Methods:**

In the 12-week main period (MP) of this double-blind, placebo-controlled study, Japanese subjects with upper-limb spasticity received one injection cycle of incobotulinumtoxinA 400 U, 250 U, or matching placebo. Eligible subjects enrolled in an open-label extension (OLEX) period of three injection cycles of incobotulinumtoxinA 400 U (32–40 weeks). The primary objective was to establish the efficacy of a single incobotulinumtoxinA injection using the Modified Ashworth Scale (MAS) wrist score. Secondary efficacy outcomes and safety were also assessed.

**Results:**

Among 100 treated subjects, AUCs for incobotulinumtoxinA 400 and 250 U were significantly different versus placebo (*p* = 0.0014 and *p* = 0.0031, respectively) for change from baseline in MAS wrist score to the end of the MP, with similar results from baseline to week 4. IncobotulinumtoxinA 400 U was superior versus placebo across other spasticity patterns and at most study visits. Improvements were maintained throughout the OLEX period. Disability Assessment Scale and Investigator’s Clinical Global Impression scores improved significantly for incobotulinumtoxinA 400 U versus placebo from baseline to week 4 (*p* = 0.0067 and *p* < 0.0001, respectively). IncobotulinumtoxinA was well tolerated up to 52 weeks, with no unexpected adverse events.

**Conclusion:**

IncobotulinumtoxinA reduced (pathologically) increased muscle tone, improved functionality and was well tolerated in Japanese subjects with post-stroke upper-limb spasticity.

## Introduction

From 1990 to 2016, the lifetime risk of stroke increased from 22.8 to 24.9% globally [1]. In East Asia, the lifetime risk of stroke increased from 29.7 to 38.8%, a much higher jump than the global estimate [1]. In Japan specifically, there has been a surge in the proportion of the population > 65 years of age in recent years, and the number of stroke patients has increased markedly in parallel. For example, in the year 2000 there were an estimated 1.7 million stroke patients in Japan, which grew to around 2.8 million in 2013 [2].

While stroke-related mortality has decreased in Japan, a phenomenon often attributed to Japan’s efficient social care system and the use of anti-hypertensive drugs—around two-thirds of stroke survivors are unable to return to their pre-illness activities [2]. The most common deficit amongst stroke survivors is motor dysfunction [3]; in particular, it has been reported that up to 42.6% of 211 patients experienced muscle spasticity after stroke [4]. Disabling or severe spasticity following the first-ever stroke occurs in approximately 2.0–15.6% of stroke survivors [4–6] and is a significant burden on patients, caregivers, and society as a whole [2]. Severe spasticity has been reported more frequently in the upper limbs compared with the lower limbs (18.9% and 5.5%, respectively) [4].

Guidelines recommend treatment with botulinum neurotoxin (BoNT) for spasticity, as part of a multidisciplinary approach to therapy, including both BoNT injections and physical therapy [7, 8]. Currently, in Japan, there is only one BoNT type A (BoNT-A) formulation, onabotulinumtoxinA, approved for the treatment of lower and upper limbs [9]. In some countries, the maximum dose for upper-limb spasticity of onabotulinumtoxinA is 240 U with at least a 12 week treatment interval, and this is restricted to the treatment of spasticity in the wrist flexors, finger flexors, and thumb flexors/adductors [10]. The treatment interval is restricted to avoid the development of neutralizing antibodies, which may be a contributing factor to secondary treatment failure [10]. Accordingly, the Japanese label permits a maximum dose of 240 U for upper-limb spasticity (and 360 U for combined upper and lower-limb spasticity) followed by potential retreatment at > 12 weeks [9]. However, there is evidence to suggest that many patients would prefer a BoNT-A treatment interval of shorter duration; in some cases, patients are clinically considered to require a higher total dose than that currently approved [11, 12]. Therefore, there is a clinical need for a higher maximum dose and flexible treatment interval.

The efficacy and safety of incobotulinumtoxinA (a BoNT-A free from complexing proteins; Merz Pharmaceuticals GmbH, Frankfurt am Main, Germany), up to a total of 500 U per injection session, has previously been established for the multi-pattern treatment of upper-limb spasticity in predominantly Caucasian subjects [13–17].

The present study (J-PURE; JapicCTI Number: CTI-153029) was the first prospective, double-blind, placebo-controlled investigation of the safety and efficacy of incobotulinumtoxinA at total doses up to 400 U in the treatment of upper-limb spasticity in a Japanese population. Here, we report the results of the 12-week main period (MP) and the open-label extension (OLEX; up to an additional 32–40 weeks).

## Methods

### Study design

This Phase III prospective, randomized, double-blind, placebo-controlled study, conducted between November 2015 and April 2018 at multiple centers in Japan, recruited subjects with post-stroke upper-limb spasticity. The study included three periods: an open-label lead-in tolerability period (LITP), a randomized, double-blind, placebo-controlled MP, and a subsequent OLEX period.

Subjects enrolled in the LITP received a single injection cycle of incobotulinumtoxinA 400 U into the muscles of the forearm and upper arm. A safety assessment of the LITP by the study sponsor and an independent Data Monitoring Committee (DMC) determined whether the dose of up to 400 U could be used in the MP and OLEX. Details of the LITP methodology and results are reported elsewhere [18].

As there were no safety concerns noted during the LITP, the sponsor followed the independent DMC’s recommendation to continue the study with incobotulinumtoxinA 400 U and 250 U. The MP was a randomized, double-blind, placebo-controlled study, with one injection cycle of incobotulinumtoxinA 400 U, incobotulinumtoxinA 250 U, or matching placebo. Subjects were randomized to receive incobotulinumtoxinA 400 U; high-dose placebo; incobotulinumtoxinA 250 U; and low-dose placebo at the ratio of 4:2:2:1, respectively.

Subjects from the MP and LITP were eligible for inclusion in the OLEX. The OLEX period comprised three injection cycles of incobotulinumtoxinA 400 U. The post-injection interval was flexible (10–14 weeks) for OLEX injection cycles 1 and 2 and fixed (12 weeks) for OLEX injection cycle 3. The incobotulinumtoxinA doses administered, and the patterns and muscle groups injected in the MP and OLEX, are summarized in Table 1.

**Table 1:** IncobotulinumtoxinA doses administered, treatment patterns and injected muscles in the MP and OLEX

	Units (high dose, 400 U)	Units (low dose, 250 U)	mL^a^	Number of injection sites^a^
MP: Upper-limb treatment, with treatment of thumb-in-palm				
Flexed wrist	100	62.50	2.0	–
Flexor carpi radialis^b^	50	31.25	1.0	1–2
Flexor carpi ulnaris^b^	50	31.25	1.0	1–2
Clenched fist	100	62.50	2.0	–
Flexor digitorum superficialis^b^	50	31.25	1.0	1–2
Flexor digitorum profundus^b^	50	31.25	1.0	1–2
Flexed elbow and pronated forearm	150	93.75	3.0	–
Biceps^b^	100	62.50	2.0	2–4
Pronator teres^b^	50	31.25	1.0	1–2
Thumb-in-palm	50	31.25	1.0	–
Flexor pollicis longus^b^	20	12.50	0.4	1
Adductor pollicis^b^	20	12.50	0.4	1
Flexor pollicis brevis or opponens pollicis^b^	10	6.25	0.2	1
MP: Upper-limb treatment, without treatment of thumb-in-palm				
Flexed wrist	100	62.50	2.0	–
Flexor carpi radialis^b^	50	31.25	1.0	1–2
Flexor carpi ulnaris^b^	50	31.25	1.0	1–2
Clenched fist	100	62.50	2.0	–
Flexor digitorum superficialis^b^	50	31.25	1.0	1–2
Flexor digitorum profundus^b^	50	31.25	1.0	1–2
Flexed elbow and pronated forearm	200	125.00	4.0	–
Biceps^b^	100	62.50	2.0	2–4
Brachialis^b^	50	31.25	1.0	1–2
Pronator teres^b^	50	31.25	1.0	1–2

OLEX: Upper-limb treatment	Units^c^	mL^c^	Number of injection sites^a^
Flexed wrist			–
Flexor carpi radialis^d^	25–100	0.5–2.0	1–2
Flexor carpi ulnaris^d^	20–100	0.4–2.0	1–2
Clenched fist	–	–	–
Flexor digitorum superficialis^d^	25–100	0.5–2.0	1–2
Flexor digitorum profundus^d^	25–100	0.5–2.0	1–2
Flexed elbow and pronated forearm	–	–	–
Brachioradialis^d^	25–100	0.5–2.0	1–3
Biceps^d^	50–200	1.0–4.0	2–4
Brachialis^d^	25–100	0.5–2.0	1–2
Pronator quadratus^d^	10–50	0.2–1.0	1
Pronator teres^d^	25–75	0.5–1.5	1–2
If thumb spasticity is present^c^	–	–	–
Flexor pollicis longus	10–50	0.2–1.0	1
Adductor pollicis	5–30	0.1–0.6	1
Flexor pollicis brevis or opponens pollicis	5–30	0.1–0.6	1

*MP* main period; *OLEX* open-label extension

^a^Maximum 1.0 mL per injection site

^b^Injection of all these muscles is mandatory

^c^If muscle is chosen for injection. For muscles not requiring treatment, 0 U/mL might be applicable

^d^400 U dose distribution at the investigator’s discretion

### Study population

Subjects were eligible for the study if they were: (i) 20–80 years of age (< 65 years of age in the LITP) with unilateral post-stroke upper-limb spasticity; (ii) botulinum toxin-naïve or pre-treated with onabotulinumtoxinA ≥ 16 weeks prior to the respective screening visit; (iii) had Modified Ashworth Scale (MAS) [19] ratings of ≥ 3 and ≥ 2 for wrist flexor and finger flexor muscle tone, respectively, at screening and baseline visits; (iv) had Disability Assessment Scale (DAS) [20] rating ≥ 2 for at least one functional disability domain at screening and baseline, and (v) had a clinical need (determined by the investigators) for a total dose of incobotulinumtoxinA 400 U. Subjects were excluded from the study if they had fixed contracture or muscle hypertonia of another type (e.g., rigidity) in the affected joint(s) to be treated, or bilateral upper-limb paresis, paralysis or tetraparesis.

### Efficacy assessments and study objectives

The primary objective of the study was to confirm, during the MP, the efficacy of a single injection cycle with incobotulinumtoxinA at two dose levels compared with matching placebo, in Japanese subjects with post-stroke upper-limb spasticity. The extent of spasticity was determined using the MAS wrist flexor score, a measure of muscle resistance during passive stretch (which relies on muscle tone) [19].

Secondary efficacy objectives were (i) to investigate the efficacy of a single injection in the MP versus placebo using the MAS spasticity score for the upper-limb muscle groups other than wrist flexors (i.e., finger flexors, thumb flexors, elbow flexors, and forearm pronators), (ii) to investigate the effect of incobotulinumtoxinA compared with placebo as measured on the DAS; and (iii) to assess the Clinical Global Impression (CGI) of patients, investigators, and caregivers. MAS assessed spasticity in five muscle groups (wrist flexors, elbow flexors, finger flexors, thumb muscles, and forearm pronators) on a 6-point scale, with 0 = no increase in muscle tone and 4 = affected part(s) rigid in flexion or extension [19]. An additional score of 1^+^ denoted a slight increase in muscle tone, manifested by a catch, followed by minimal resistance throughout the remainder (less than half) of the range of movement [19] and this was defined as “1.5” for analysis purposes.

DAS was utilized to determine the effect of upper-limb spasticity on activities of daily living [20]. Using DAS, the extent of functional impairment was assessed on a 4-point scale from 0 (no disability) to 3 (severe disability, normal activities limited) across four domains (hygiene, dressing, limb position, and pain); for each subject, one domain was identified as the principal target domain at screening and baseline of each injection cycle. CGI was measured on an 11-point Likert scale from − 5 (worst possible status) to + 5 (best possible status).

Additional post-hoc analyses evaluated MAS during the OLEX by injection cycle length of the first and second OLEX injection cycles (10 weeks [67–73 days], > 10–12 weeks [74–87 days], > 12–14 weeks [88–101 days]) to assess the efficacy of different re-injection intervals.

### Safety assessments and study objectives

Additional secondary objectives were to investigate the safety of incobotulinumtoxinA 400 U and 250 U compared with placebo, and the safety of repeated doses of incobotulinumtoxinA for a total treatment duration of up to 52 weeks. Lists of adverse events (AEs), severity, and relationship to the study treatment were recorded, with AEs coded according to the Medical Dictionary for Regulatory Activities (MedDRA) version 20.1, which was valid at the time point of database closure. A post hoc analysis was conducted to assess AEs based on the length of the first and second OLEX injection cycles (10 weeks, > 10–12 weeks, > 12–14 weeks). Serious AEs included those that were life-threatening, required hospitalization, or resulted in death. Subjects were actively questioned and closely monitored for signs of potential toxin spread as indicated by specific ‘AEs of special interest’, such as swallowing difficulties, speech or breathing disorders, diplopia, or muscular weakness. Seizure was defined as an indication-specific AE for close monitoring and subjects were asked at each visit if this event had occurred since last contact. Standard safety physical and laboratory assessments were performed at each visit, or according to the study schedule. Blood samples were drawn for BoNT-A antibodies at baseline and end-of-study visits.

### Statistical analyses

The primary efficacy variable was the area under the curve (AUC) of the change from baseline in the MAS wrist score to the end of the MP (week 12). A confirmatory analysis based on the full analysis set (FAS) was performed hierarchically. The MP FAS comprised all subjects in the safety evaluation set (SES) for whom a baseline value of MAS for wrist assessment was available. A comparison of incobotulinumtoxinA 400 U and the high-dose placebo groups was first carried out, then if significant results were observed from this comparison, a confirmatory comparison between incobotulinumtoxinA 250 U and low-dose placebo groups was performed. An analysis of covariance (ANCOVA) was applied, in which baseline MAS wrist score was the covariate with pooled site, with treatment (400 U or 250 U vs. matching placebo) and gender as factors (*α* = 0.05, two-sided tests). Isolated missing values were calculated using non-missing values, and any remaining missing values were imputed from baseline wrist MAS (baseline observation carried forward; BOCF). A sensitivity analysis was also performed on the per-protocol set (PPS), including those subjects in the FAS who had no significant protocol deviations. Descriptive statistics by treatment group, including least square (LS) means, *p*-values and confidence limits by total population and ANCOVA level, were also recorded.

The secondary efficacy variable, change in the MAS wrist score from baseline (day 1) to week 4, was analyzed using the same model as for the primary efficacy analyses and the BOCF imputation method. Descriptive analyses, based on the FAS and PPS populations, were also performed for the secondary efficacy variable.

Other efficacy variables were analyzed descriptively. Exploratory ANCOVA with baseline as covariate and with gender, site, and treatment as factors was applied similarly to the primary and secondary efficacy analyses. No statistical tests were performed on data from the LITP and OLEX periods.

Safety analyses were performed on the SES, comprising all patients who were exposed to incobotulinumtoxinA during each respective study period. Standard laboratory values, antibody data, vital signs, body weight, and physical examination variables were analyzed descriptively and screened for individual clinically relevant values and changes from baseline, where applicable.

## Results

### Subjects

**Fig. 1:**
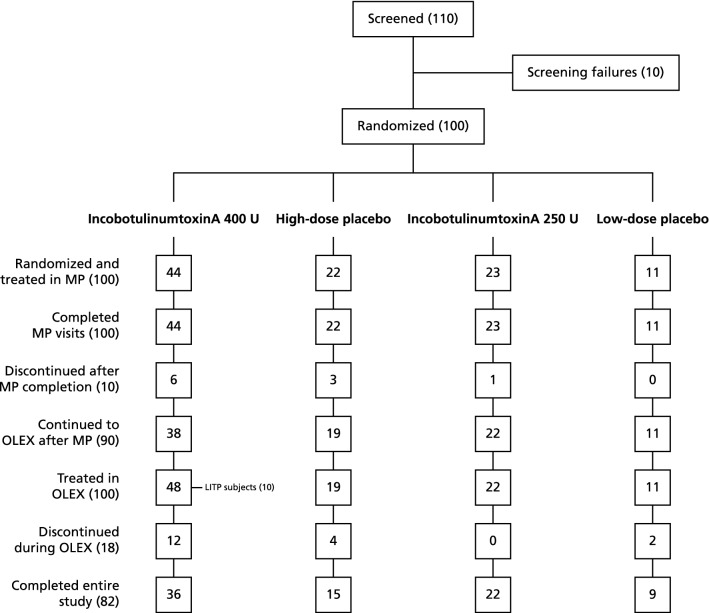
Subject disposition in MP and OLEX. *MP* main period, *OLEX* open-label extension, *LITP* lead-in treatment period

The study was conducted at 32 sites in Japan, 31 of which enrolled subjects. In total, 100 subjects were randomized and treated in the MP (incobotulinumtoxinA 400 U, *n* = 44; high-dose placebo, *n* = 22; incobotulinumtoxinA 250 U, *n* = 23; low-dose placebo, *n* = 11) and were included in the FAS and SES of the MP. Of these subjects, 90 completed the MP and entered the OLEX (incobotulinumtoxinA 400 U, *n* = 38; high-dose placebo, *n* = 19; incobotulinumtoxinA 250 U, *n* = 22; low-dose placebo, *n* = 11). Overall, 100 subjects (FAS and SES), including 10 subjects from the LITP, entered the OLEX, and 82 subjects completed all four cycles of the study (Fig. 1).

Subject baseline demographics are summarized in Table 2. Briefly, all subjects were of Asian race, and there were no differences between the treatment groups in gender ratio, mean age, weight or body mass index (BMI).

**Table 2:** Subject demographics in the MP

	Treatment group				
	High-dose		Low-dose		Total (*N* = 100)
	IncobotulinumtoxinA (*N* = 44)	Placebo (*N* = 22)	IncobotulinumtoxinA (*N* = 23)	Placebo (*N* = 11)	
Mean age, years (SD)	59.8 (11.3)	54.7 (14.2)	62.8 (9.8)	62.6 (9.5)	59.7 (11.7)
Gender, *n* (%)					
Male	34 (77.3)	15 (68.2)	18 (78.3)	8 (72.7)	75 (75.0)
Female	10 (22.7)	7 (31.8)	5 (21.7)	3 (27.3)	25 (25.0)
Race, *n* (%)					
Asian	44 (100)	22 (100)	23 (100)	11 (100)	100 (100)
Mean weight, kg (SD)	63.9 (11.3)	66.0 (15.2)	67.9 (9.5)	62.1 (9.5)	65.1 (11.7)
Mean BMI, kg/m^2^ (SD)	24 (3)	25 (5)	25 (4)	23 (3)	24 (4)

*BMI* body mass index, *SD* standard deviation

### Efficacy

#### Muscle tone

**Fig. 2:**
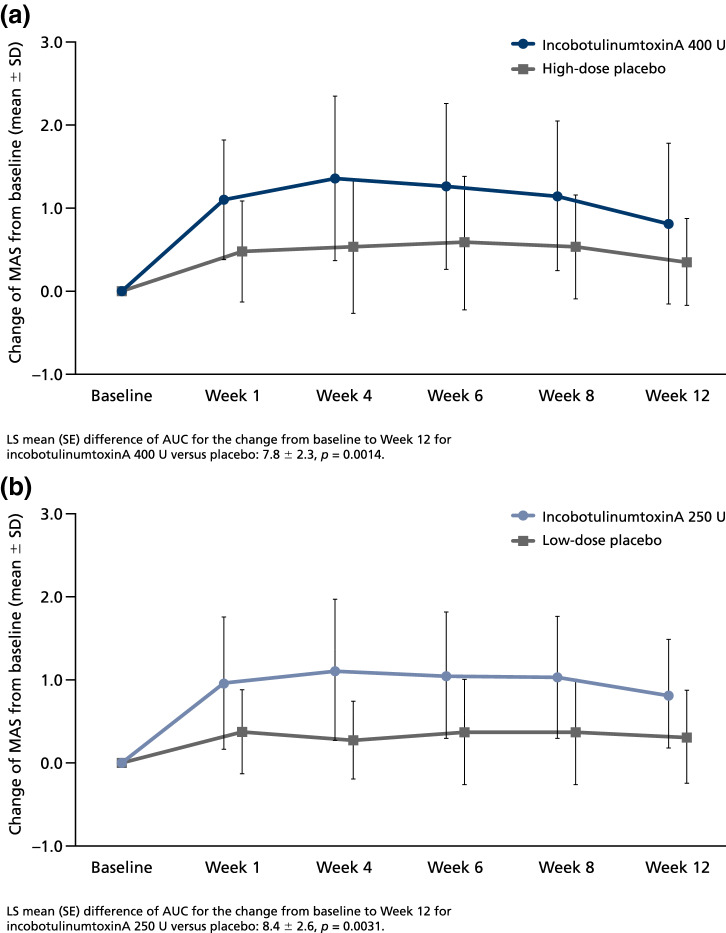
Time course of MAS wrist score change from baseline to the end of the MP (Week 12) for **a** incobotulinumtoxinA 400 U, and **b** incobotulinumtoxinA 250 U versus placebo. ANCOVA of AUC for change from baseline in MAS wrist score (high-dose placebo, *n* = 11; incobotulinumtoxinA 400 U, *n* = 23; low-dose placebo, *n* = 11; incobotulinumtoxinA 250 U, *n* = 23). *ANCOVA* analysis of covariance, *AUC* area under the curve, *LS* least squares, *MAS* Modified Ashworth Scale, *SD* standard deviation, *SE* standard error

For the primary efficacy variable of AUC for the changes in MAS wrist score from baseline to the end of the MP, both doses of incobotulinumtoxinA (400 U and 250 U) showed a statistically significant difference versus matching placebo (week 12), indicating the superiority of incobotulinumtoxinA over placebo (Fig. 2). LS mean differences of AUC (SE) [95% confidence intervals, CI] in favor of treatment with incobotulinumtoxinA compared with placebo were 7.75 (2.322) [3.10–12.39] for the 400 U dose group (*p* = 0.0014) and 8.35 (2.593) [3.05–13.64] for the 250 U dose group (*p* = 0.0031) (Fig. 2). In the high-dose groups, mean AUC for the change in baseline MAS was consistently greater in the incobotulinumtoxinA group than the placebo group regardless of gender, pre-treatment or study site. Results from the sensitivity analysis performed in the PPS population were consistent with results for the FAS, in that the differences between incobotulinumtoxinA and placebo were significant (*p* = 0.0016 and *p* = 0.0041 for the high- and low-dose groups, respectively).

**Fig. 3:**
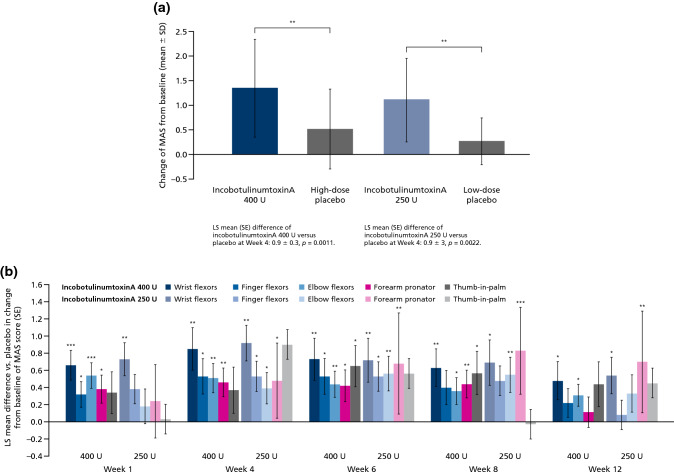
Change from baseline in MAS scores for incobotulinumtoxinA versus placebo **a** for wrist flexors at week 4, and **b** additional clinical patterns up to week 12 of the MP. *p ≤ 0.05; ** *p* ≤ 0.01; *** *p* ≤ 0.001. ANCOVA of change in MAS wrist score from baseline to week 4 (**a**) and by clinical pattern and visit in MP (high-dose placebo, *n* = 22; incobotulinumtoxinA 400 U, *n* = 44; low-dose placebo, *n* = 11; incobotulinumtoxinA 250 U, *n* = 23). *LS* least squares, *MAS* Modified Ashworth Scale, *MP* main period, *SD* standard deviation, *SE* standard error

For the secondary efficacy variable, both doses of incobotulinumtoxinA (400 U and 250 U) showed significant changes versus placebo in wrist MAS score from baseline to week 4 (Fig. 3a) in the BOCF and FAS populations. LS mean differences (SE) [95% CI] in favor of treatment with incobotulinumtoxinA compared with placebo were 0.85 (0.248) [0.35–1.35] for the 400 U dose group (*p* = 0.0011), and 0.92 (0.276) [0.36–1.49] for the 250 U dose group (*p* = 0.0022). Results from the sensitivity analysis performed in the PPS population were consistent with results of the FAS, whereby the differences between incobotulinumtoxinA and placebo were statistically significant (*p* = 0.0013 and *p* = 0.0030 for the high- and low-dose groups, respectively).

Analysis of MAS scores for finger and elbow flexors and forearm pronators showed that 400 U incobotulinumtoxinA had a pronounced, significant effect versus placebo across most treated clinical spasticity patterns and at most of the study visits, and a significant transient effect on thumb-in-palm. A dose of 250 U incobotulinumtoxinA resulted in delayed or transient significant effects on finger flexor, elbow flexor, and forearm pronator MAS scores (Fig. 3b).

For the incobotulinumtoxinA dose of 400 U, the percentage of responders (subjects with ≥ 1-point reduction in MAS score) was higher in the treatment group versus the placebo group: *p*-values for treatment comparisons were < 0.05 for flexed wrist (responder rate range: 75.0–81.8% [treatment group] and 36.4–45.5% [placebo group] at weeks 1–8), clenched fist (responder rate range: 68.2–70.5% [treatment group] and 27.3–36.4% [placebo group] at weeks 4–6) and flexed elbow (responder rate range: 34.1–56.8% [treatment group] and 9.1–22.7% [placebo group] at weeks 1–6 and week 12). Correspondingly, for an incobotulinumtoxinA dose of 250 U, the percentage of responders was higher in the treatment group relative to the placebo group: *p*-values for treatment comparisons were < 0.05 for flexed wrist (responder rate range: 65.2–69.6% [treatment group] and 27.3–36.4% [placebo group] at weeks 1–12), clenched fist (responder rate: 52.2% [treatment group] and 18.2% [placebo group] at week 4) and for flexed elbow (responder rate range: 43.5–56.5% [treatment group] and 18.2–27.3% [placebo group] at weeks 4–8).

In the OLEX, the mean (SD) changes in MAS wrist score from study baseline to week 4/end of injection cycle were 1.43 (0.78)/0.88 (0.69) for cycle 2, 1.49 (0.74)/1.01 (0.69) for cycle 3 and 1.50 (0.69)/1.22 (0.72) for cycle 4. Mean changes in MAS wrist scores from study baseline to week 4 of injection cycle 2 were comparable irrespective of the treatments given in the LITP/MP: 1.49 (0.82) for incobotulinumtoxinA 400 U, 1.53 (0.79) for high-dose placebo, 1.32 (0.80) for incobotulinumtoxinA 250 U, and 1.23 (0.52) for low-dose placebo.

Similar changes in MAS scores were obtained for finger, elbow, and forearm pronator in the OLEX period, whereas the changes in MAS thumb flexor scores were smaller in magnitude (data not shown).

Decreases in mean MAS, indicating an improvement, were seen in all three subgroups by length of injection cycle (10 weeks [incobotulinumtoxinA 400 U, *n* = 27; high-dose placebo, *n* = 11; incobotulinumtoxinA 250 U, *n* = 13; low-dose placebo, *n* = 4], > 10–12 weeks [incobotulinumtoxinA 400 U, *n* = 4; high-dose placebo, *n* = 2; incobotulinumtoxinA 250 U, *n* = 2; low-dose placebo, *n* = 1] and > 12–14 weeks [incobotulinumtoxinA 400 U, *n* = 3; high-dose placebo, *n* = 2; incobotulinumtoxinA 250 U, *n* = 1; low-dose placebo, *n* = 0]) for most time points of all clinical patterns. The magnitude of decreases was similar between the subgroups by injection cycles for most time points of all clinical patterns.

#### Functionality

**Fig. 4:**
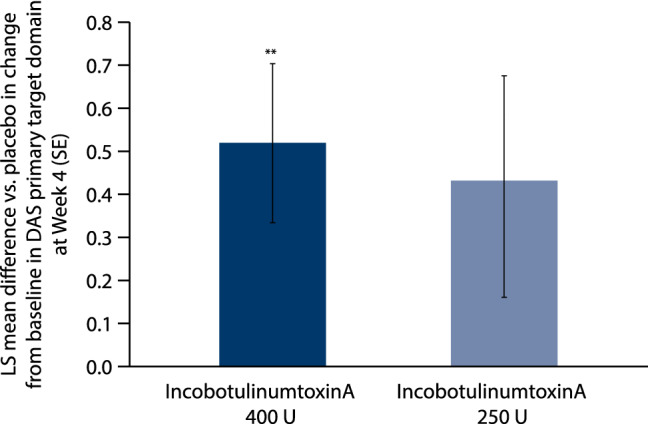
Change from baseline in DAS scores of the principal therapeutic target domain for incobotulinumtoxinA versus placebo at week 4 of the MP. ** *p* ≤ 0.01. ANCOVA of change in DAS score from baseline to week 4 (incobotulinumtoxinA 400 U, *n* = 44; incobotulinumtoxinA 250 U, *n* = 23). *DAS* disability assessment scale, *LS* least squares, *MP* main period, *SE* standard error

In the MP, a significant change versus placebo was observed in the DAS score of the principal therapeutic target domains (defined as limb position for 43.0% of subjects, hygiene for 34.0% of subjects, and dressing for 23.0% of subjects at screening; no subjects had pain as their principal therapeutic target domain) from baseline to week 4 of the MP in the high-dose group of incobotulinumtoxinA, but not the low-dose group (Fig. 4). LS mean difference [SE] versus placebo was significantly greater with incobotulinumtoxinA 400 U at week 4 (0.52 [0.184]; *p* = 0.0067), week 6 (0.53 [0.198]; *p* = 0.0097) and week 8 (0.40 [0.196]; *p* = 0.0476). The change versus placebo was greater with incobotulinumtoxinA 250 U at week 8 (0.53 [0.226]; *p* = 0.0254). Mean [SD] change from baseline for incobotulinumtoxinA 400 U was 0.73 [0.79], 0.75 [0.78], and 0.70 [0.82] at weeks 4, 6, and 8, respectively, and for 250 U was 0.61 [0.66] at week 8.

In the OLEX period, the mean (SD) changes in the DAS score of the principal therapeutic target domain from study baseline to week 4 of injection cycles 2, 3 and 4 were 0.78 (0.83), 0.94 (0.87), and 0.99 (0.91), respectively. When considering the treatment received during the MP, mean (SD) changes in DAS scores of the principal therapeutic target domain from study baseline to week 4 of injection cycle 2 (OLEX) were numerically greater in subjects who had received incobotulinumtoxinA during the MP than in those who had received placebo, but were comparable between the incobotulinumtoxinA 400 U and 250 U dose groups (0.83 [0.84] for incobotulinumtoxinA 400 U, 0.58 [0.90] for high-dose placebo, 1.00 [0.76] for incobotulinumtoxinA 250 U, and 0.45 [0.69] for low-dose placebo).

A significant change in investigators’ CGI assessment at week 4 of the MP was observed with incobotulinumtoxinA 400 U compared to placebo, but this change was not seen with 250 U***.*** The change versus placebo was numerically greater (indicating greater improvement) for incobotulinumtoxinA 400 U at week 12 (LS mean difference [CI]: − 1.37 [− 2.30 to − 0.43]. In the OLEX period, the mean (SD) changes in investigators’ CGI from study baseline to week 4 of injection cycles 2, 3 and 4 were − 2.67 (2.35), − 3.18 (2.12), and − 3.36 (2.23), respectively.

A significant change in patients’ CGI assessment at week 4 (LS mean difference [CI]: − 1.53 [− 2.34 to 0.73]; *p* = 0.0003) and week 12 (LS mean difference [CI]: − 1.34 [− 2.15 to − 0.54]) of the MP was observed with incobotulinumtoxinA 400 U compared with placebo, and with incobotulinumtoxinA 250 U compared with placebo at week 12 only (LS mean difference [CI] − 1.80 [− 3.34 to − 0.26]). In the OLEX period, the mean (SD) changes in patients’ CGI from study baseline to week 4 of injection cycles 2, 3 and 4 were − 2.24 (2.18), − 2.25 (2.12), and − 2.68 (2.41), respectively.

### Safety

#### Main period

AEs are summarized in Table 3. Overall, incobotulinumtoxinA (400 U and 250 U) was well tolerated, and no unexpected AEs were observed. The incidence of AEs was overall comparable in the incobotulinumtoxinA 400 U and 250 U dose groups, and there were no treatment-related AEs leading to discontinuation.

**Table 3:** Overall summary of AEs in the (a) MP and (b) OLEX periods

(a) MP (*n*, %)	Treatment group					
	High-dose		Low-dose		Total	
	IncobotulinumtoxinA (*N* = 44)	Placebo (*N* = 22)	IncobotulinumtoxinA (*N* = 23)	Placebo (*N* = 11)	IncobotulinumtoxinA (*N* = 67)	Placebo (*N* = 33)
AE	22 (50.0)	8 (36.4)	10 (43.5)	4 (36.4)	32 (47.8)	12 (36.4)
AE related to treatment	3 (6.8)	0	2 (8.7)	0	5 (7.5)	0
Serious AEs	1 (2.3)	0	1 (4.3)	1 (9.1)	2 (3.0)	1 (3.0)
Serious AEs related to treatment	0	0	0	0	0	0
AE of special interest	2 (4.5)	0	1 (4.3)	0	3 (4.5)	0
AE of special interest related to treatment	2 (4.5)	0	0	0	2 (3.0)	0
AE leading to discontinuation	1 (2.3)	2 (9.1)	0	0	1 (1.5)	2 (6.1)
AE leading to discontinuation related to treatment	0	0	0	0	0	0
Deaths	0	0	0	0	0	0

(b) OLEX (*n*, %)	Cycle 2 (*N* = 100)	Cycle 3 (*N* = 91)	Cycle 4 (*N* = 82)	Total (*N* = 100)
AE	36 (36.0)	30 (33.0)	21 (25.6)	65 (65.0)
AE related to treatment	3 (3.0)	0	3 (3.7)	6 (6.0)
Serious AEs	1 (1.0)	3 (3.3)	0	4 (4.0)
Serious AEs related to treatment	0	0	0	0
AE of special interest	2 (2.0)	2 (2.2)	0	4 (4.0)
AE of special interest related to treatment	2 (2.0)	0	0	2 (2.0)
AE leading to discontinuation	1 (1.0)	1 (1.1)	0	2 (2.0)
AE leading to discontinuation related to treatment	0	0	0	0
Deaths	0	0	0	0

*AE* adverse event, *MP* main period, *n* number of subjects, *OLEX* open-label extension

In the incobotulinumtoxinA 400 U and 250 U dose groups, two subjects (8.7%) and three subjects (6.8%) experienced AEs considered related to treatment, respectively. There were three subjects with a total of five AEs of special interest; four events in two (4.5%) subjects receiving incobotulinumtoxinA 400 U (dysarthria, *n* = 2; dysphagia, *n* = 1; muscular weakness, *n* = 1) and one event (4.3%) in a subject receiving incobotulinumtoxinA 250 U (constipation, *n* = 1). All AEs in the 400 U dose group were considered to be related to treatment. The AE in the 250 U dose group was not considered to be related to treatment. All AEs were mild in intensity and resolved without any intervention.

For three subjects, three serious AEs were reported, one each for the incobotulinumtoxinA 400 U (polymyalgia rheumatica), 250 U (femur fracture), and low-dose placebo groups (cholelithotomy); none were considered to be treatment-related. No fatal AEs occurred during the MP.

#### Open-label extension period

IncobotulinumtoxinA continued to be well tolerated for up to 52 weeks, over the three additional treatment cycles of the OLEX period; there were no deaths in any period of the study and AEs were comparable overall in frequency and severity across the two dose groups (Table 3).

In total, six subjects (6.0%) experienced AEs considered related to treatment, three each in cycle 2 and cycle 4. There were no treatment-related AEs leading to discontinuation. Four subjects experienced five AEs of special interest (muscular weakness, *n* = 2 [1 mild and 1 moderate in intensity]; moderate hypotonia, *n* = 1; moderate dyspnea, *n* = 1, and moderate accommodation disorder, *n* = 1), two of which were considered treatment-related, and all were resolved with the exception of the accommodation disorder event. Five serious treatment-emergent AEs in the OLEX period were reported in four subjects. None of the serious treatment-emergent AEs were considered to be related to treatment, and no fatal AEs occurred during the OLEX period. From baseline to the end-of-cycle visits, there were no relevant changes in clinical laboratory values, vital signs, or ECG.

For most subjects (*n* = 55) the interval of the first and second injection cycles of the OLEX was 10 weeks; among these, 35 reported AEs and, in 3 of these subjects, the AEs were considered to be treatment-related (1 subject in the incobotulinumtoxinA 400 U group and 2 subjects in the 250 U group). Seven (out of nine) subjects and five (out of six) subjects with a cycle length of > 10–12 and > 12–14 weeks for the first and second OLEX injection cycles, respectively, exhibited AEs and none of these AEs were considered to be treatment-related.

Five subjects were found to be positive for anti-BoNT/A antibodies at baseline, and all of these subjects had previously been treated with BoNT. According to the fluorescence immunoassay for antibodies, seven subjects were positive for anti-BoNT/A antibodies at the end-of-study visit: five of those subjects had previously been positive at baseline (and week 12) whereas two subjects were newly positive. However, these two subjects were found to be negative for neutralizing anti-BoNT/A antibodies using the hemidiaphragm assay (HDA) method. There was no indication of secondary non-response in any of the subjects with neutralizing antibodies at the end of study.

## Discussion

This study shows that treatment with a single injection of incobotulinumtoxinA (either 400 U or 250 U) versus placebo in Japanese subjects with multi-pattern, post-stroke upper-limb spasticity, was effective in reducing (pathologically) increased muscle tone, as measured by MAS scores for the wrist at week 12 (primary efficacy variable), and at week 4. Observed improvements in wrist spasticity were also reported in a previous study in which 200–240 U onabotulinumtoxinA was administered to the wrist (100 U), finger (100 U), and thumb (40 U, optional additional dose) in Asian subjects [21]. Furthermore, upper-limb muscle tone improvements have been observed, primarily in Caucasian populations, following treatment with incobotulinumtoxinA doses up to 400 U per injection cycle for post-stroke upper-limb spasticity: however, these studies utilized the Ashworth Scale instead of the MAS [13–16] so efficacy outcomes are not directly comparable to that of the current study.

Distribution of the total dose of BoNT-A in the present study was more flexible than in a similar earlier study in Asian subjects [21], with the incobotulinumtoxinA 400 U dose enabling treatment of a greater number of clinical patterns where required. In addition to improvements in muscle tone of the wrist, the broad distribution of incobotulinumtoxinA 400 U resulted in improvement in spastic muscle tone in most other upper-limb muscle groups, i.e., finger flexors, elbow flexors, and forearm pronators and corresponding clinical patterns, at the majority of study visits.

Improvements in muscle tone were accompanied by significant improvements in functionality, as evidenced by the changes observed in DAS score for the principal domain, and in the investigators’ and patients’ CGI scores from baseline to week 4, with incobotulinumtoxinA 400 U versus placebo in the MP, but not with the 250 U dose. These results suggest a possible dose–response relationship, and a more pronounced benefit with the higher dose, consistent with significant improvements in MAS wrist and CGI scores with a higher dose of onabotulinumtoxinA, and variability in DAS, shown previously in Asian subjects [21].

Overall, incobotulinumtoxinA doses up to 400 U were well tolerated for the treatment of post-stroke upper-limb spasticity in Japanese subjects for up to 52 weeks, and over four treatment cycles. No subjects died during the study, and no unexpected AEs were reported. Notably, no subjects discontinued the study due to treatment-related AEs.

In general, the longer-term safety profile of incobotulinumtoxinA in Asian subjects was similar to that of Caucasian subjects with upper-limb spasticity treated with incobotulinumtoxinA up to 400 U in previously published studies in Europe and the United States [13, 14, 16, 22]; the incidence of treatment-related AEs occurred at a similar low frequency in all studies, and the incidence of AEs and treatment-related AEs in the present study was similar to that of Asian subjects treated with onabotulinumtoxinA [21].

In the current study, treatment-related AEs, as well as AEs of special interest, were only reported with incobotulinumtoxinA treatment. However, there were no notable differences between the treatment groups in the incidences of serious AEs and, among the AEs of special interest, only muscle weakness was reported in both the MP and OLEX periods, consistent with the mechanism of action and known side-effect profile of BoNT formulations including incobotulinumtoxinA [17, 23]. Furthermore, there was no tendency towards an increased incidence of AEs associated with repeated administrations of incobotulinumtoxinA in the OLEX period, and no serious AEs were considered to be related to treatment. All subjects that were positive for anti-BoNT/A antibodies at baseline had been treated with BoNT before study enrollment. Notably, there was no indication of the development of secondary non-response in any of the subjects and, according to the HDA method, no subjects newly developed neutralizing antibodies during this study.

### Strengths and limitations

The strengths of this study included the double-blind, placebo-controlled, randomized nature of the MP design, and the flexible re-injection interval during cycles 1 and 2 of the OLEX period, to better mirror real-world clinical practice. Furthermore, the broad administration of incobotulinumtoxinA 400 U across various clinical patterns of spasticity led to improvements in muscle tone and was also reflected by improvements in patient-related outcomes (DAS; patients’ CGI). A limitation of the OLEX part of the study was that, although injection cycle 1 was blinded and placebo-controlled, injection cycles 2 to 4 were open-labelled (with all participants receiving the active treatment); as such, the potential for bias in efficacy outcome assessments in these cycles could not be fully ruled out. The open-labelled nature of injection cycles 2 to 4 permitted varied doses to be administered per muscle depending on the patient’s individual clinical requirement.

## Conclusion

IncobotulinumtoxinA doses of up to 400 U were shown to be effective in Japanese subjects with multi-pattern, post-stroke upper-limb spasticity in terms of improved muscle tone and functionality. Although both 250 U and 400 U had therapeutic benefits, the efficacy of incobotulinumtoxinA was more pronounced with the higher dose. For both doses (250 U and 400 U), the drug was well tolerated with no safety concerns.

## References

[1] Feigin VL, Nguyen G, Cercy K, Johnson CO, Alam T, Parmar PG, Abajobir AA, Abate KH, Abd-Allah F, Abejie AN, Abyu GY, Ademi Z, Agarwal G, Ahmed MB, Akinyemi RO, Al-Raddadi R, Aminde LN, Amlie-Lefond C, Ansari H, Asayesh H, Asgedom SW, Atey TM, Ayele HT, Banach M, Banerjee A, Barac A, Barker-Collo SL, Barnighausen T, Barregard L, Basu S, Bedi N, Behzadifar M, Bejot Y, Bennett DA, Bensenor IM, Berhe DF, Boneya DJ, Brainin M, Campos-Nonato IR, Caso V, Castaneda-Orjuela CA, Rivas JC, Catala-Lopez F, Christensen H, Criqui MH, Damasceno A, Dandona L, Dandona R, Davletov K, de Courten B, deVeber G, Dokova K, Edessa D, Endres M, Faraon EJA, Farvid MS, Fischer F, Foreman K, Forouzanfar MH, Gall SL, Gebrehiwot TT, Geleijnse JM, Gillum RF, Giroud M, Goulart AC, Gupta R, Gupta R, Hachinski V, Hamadeh RR, Hankey GJ, Hareri HA, Havmoeller R, Hay SI, Hegazy MI, Hibstu DT, James SL, Jeemon P, John D, Jonas JB, Jozwiak J, Kalani R, Kandel A, Kasaeian A, Kengne AP, Khader YS, Khan AR, Khang YH, Khubchandani J, Kim D, Kim YJ, Kivimaki M, Kokubo Y, Kolte D, Kopec JA, Kosen S, Kravchenko M, Krishnamurthi R, Kumar GA, Lafranconi A, Lavados PM, Legesse Y, Li Y, Liang X, Lo WD, Lorkowski S, Lotufo PA, Loy CT, Mackay MT, Abd El Razek HM, Mahdavi M, Majeed A, Malekzadeh R, Malta DC, Mamun AA, Mantovani LG, Martins SCO, Mate KK, Mazidi M, Mehata S, Meier T, Melaku YA, Mendoza W, Mensah GA, Meretoja A, Mezgebe HB, Miazgowski T, Miller TR, Ibrahim NM, Mohammed S, Mokdad AH, Moosazadeh M, Moran AE, Musa KI, Negoi RI, Nguyen M, Nguyen QL, Nguyen TH, Tran TT, Nguyen TT, Anggraini Ningrum DN, Norrving B, Noubiap JJ, O'Donnell MJ, Olagunju AT, Onuma OK, Owolabi MO, Parsaeian M, Patton GC, Piradov M, Pletcher MA, Pourmalek F, Prakash V, Qorbani M, Rahman M, Rahman MA, Rai RK, Ranta A, Rawaf D, Rawaf S, Renzaho AM, Robinson SR, Sahathevan R, Sahebkar A, Salomon JA, Santalucia P, Santos IS, Sartorius B, Schutte AE, Sepanlou SG, Shafieesabet A, Shaikh MA, Shamsizadeh M, Sheth KN, Sisay M, Shin MJ, Shiue I, Silva DAS, Sobngwi E, Soljak M, Sorensen RJD, Sposato LA, Stranges S, Suliankatchi RA, Tabares-Seisdedos R, Tanne D, Nguyen CT, Thakur JS, Thrift AG, Tirschwell DL, Topor-Madry R, Tran BX, Nguyen LT, Truelsen T, Tsilimparis N, Tyrovolas S, Ukwaja KN, Uthman OA, Varakin Y, Vasankari T, Venketasubramanian N, Vlassov VV, Wang W, Werdecker A, Wolfe CDA, Xu G, Yano Y, Yonemoto N, Yu C, Zaidi Z, El Sayed Zaki M, Zhou M, Ziaeian B, Zipkin B, Vos T, Naghavi M, Murray CJL, Roth GA, Global Burden of Disease Lifetime Risk of Stroke Collaborators (2018). Global, Regional, and Country-Specific Lifetime Risks of Stroke, 1990 and 2016. N Engl J Med.

[2] Kaji R (2015). Asian neurology and stroke. Neurology.

[3] Lawrence ES, Coshall C, Dundas R, Stewart J, Rudd AG, Howard R, Wolfe CD (2001). Estimates of the prevalence of acute stroke impairments and disability in a multiethnic population. Stroke.

[4] Urban PP, Wolf T, Uebele M, Marx JJ, Vogt T, Stoeter P, Bauermann T, Weibrich C, Vucurevic GD, Schneider A, Wissel J (2010). Occurrence and clinical predictors of spasticity after ischemic stroke. Stroke.

[5] Lundström E, Smits A, Terént A, Borg J (2010). Time-course and determinants of spasticity during the first six months following first-ever stroke. J Rehabil Med.

[6] Wissel J, Manack A, Brainin M (2013). Toward an epidemiology of poststroke spasticity. Neurology.

[7] Simpson DM, Hallett M, Ashman EJ, Comella CL, Green MW, Gronseth GS, Armstrong MJ, Gloss D, Potrebic S, Jankovic J, Karp BP, Naumann M, So YT, Yablon SA (2016). Practice guideline update summary: botulinum neurotoxin for the treatment of blepharospasm, cervical dystonia, adult spasticity, and headache: report of the Guideline Development Subcommittee of the American Academy of Neurology. Neurology.

[8] Wissel J, Ward AB, Erztgaard P, Bensmail D, Hecht MJ, Lejeune TM, Schnider P, Altavista MC, Cavazza S, Deltombe T, Duarte E, Geurts AC, Gracies JM, Haboubi NHJ, Juan FJ, Kasch H, Kätterer C, Kirazli Y, Manganotti P, Parman Y, Paternostro-Sluga T, Petropoulou K, Prempeh R, Rousseaux M, Slawek J, Tieranta N (2009). European consensus table on the use of botulinum toxin type A in adult spasticity. J Rehabil Med.

[9] Japan GKK (2018) Botox^®^ Japanese product information. https://gskpro.com/content/dam/global/hcpportal/ja_JP/products-info/botox/botox-if.pdf. Accessed 30 Aug 2019

[10] Allergan Ltd. (2017) Botox^®^ 100 U summary of product characteristics. https://www.medicines.org.uk/EMC/medicine/112/SPC/. Accessed 2 Oct 2018

[11] Wissel J, Bensmail D, Ferreira JJ, Molteni F, Satkunam L, Moraleda S, Rekand T, McGuire J, Scheschonka A, Flatau-Baque B, Simon O, Rochford ET, Dressler D, Simpson DM (2017). Safety and efficacy of incobotulinumtoxinA doses up to 800 U in limb spasticity: the TOWER study. Neurology.

[12] Bensmail D, Hanschmann A, Wissel J (2014). Satisfaction with botulinum toxin treatment in post-stroke spasticity: results from two cross-sectional surveys (patients and physicians). J Med Econ.

[13] Elovic EP, Munin MC, Kaňovský P, Hanschmann A, Hiersemenzel R, Marciniak C (2016). Randomized, placebo-controlled trial of incobotulinumtoxinA for upper-limb post-stroke spasticity. Muscle Nerve.

[14] Marciniak C, Munin MC, Brashear A, Rubin BS, Patel AT, Slawek J, Hanschmann A, Hiersemenzel R, Elovic EP (2019). IncobotulinumtoxinA efficacy and safety in adults with upper-limb spasticity following stroke: results from the open-label extension period of a phase 3 study. Adv Ther.

[15] Kaňovský P, Slawek J, Denes Z, Platz T, Sassin I, Comes G, Grafe S (2009). Efficacy and safety of botulinum neurotoxin NT 201 in poststroke upper limb spasticity. Clin Neuropharmacol.

[16] Kaňovský P, Slawek J, Denes Z, Platz T, Comes G, Grafe S, Pulte I (2011). Efficacy and safety of treatment with incobotulinum toxin A (botulinum neurotoxin type A free from complexing proteins; NT 201) in post-stroke upper limb spasticity. J Rehabil Med.

[17] Merz Pharma UK Ltd. (2017) XEOMIN^®^ (50/100/200) summary of product characteristics. https://www.medicines.org.uk/emc/medicine/24582. Accessed 17 May 2019

[18] Kagaya H, Masakado Y, Saitoh E, Fujiwara T, Abo M, Izumi S, Nodera H, Dekundy A, Hiersemenzel R, Nalaskowski C, Hanschmann A, Kaji R (2018). Safety and tolerability of incobotulinumtoxinA for the treatment of upper and lower limb spasticity in Japanese subjects. Toxicon.

[19] Bohannon RW, Smith MB (1987). Interrater reliability of a modified Ashworth scale of muscle spasticity. Phys Ther.

[20] Brashear A, Zafonte R, Corcoran M, Galvez-Jimenez N, Gracies JM, Gordon MF, McAfee A, Ruffing K, Thompson B, Williams M, Lee CH, Turkel C (2002). Inter- and intrarater reliability of the Ashworth Scale and the Disability Assessment Scale in patients with upper-limb poststroke spasticity. Arch Phys Med Rehabil.

[21] Kaji R, Osako Y, Suyama K, Maeda T, Uechi Y, Iwasaki M, GSK1358820 Spasticity Study Group (2010). Botulinum toxin type A in post-stroke upper limb spasticity. Curr Med Res Opin.

[22] Kaňovský P, Bares M, Severa S, Richardson A (2009). Long-term efficacy and tolerability of 4-monthly versus yearly botulinum toxin type A treatment for lower-limb spasticity in children with cerebral palsy. Dev Med Child Neurol.

[23] Merz Pharmaceuticals LLC (2019) Highlights of prescribing information—Xeomin^®^. https://www.accessdata.fda.gov/drugsatfda_docs/label/2019/125360s074lbl.pdf. Accessed 17 May 2019

